# Impact of Hypoxia on Drug Resistance and Growth Characteristics of *Mycobacterium tuberculosis* Clinical Isolates

**DOI:** 10.1371/journal.pone.0166052

**Published:** 2016-11-11

**Authors:** Zhonghua Liu, Yulu Gao, Hua Yang, Haiyang Bao, Lianhua Qin, Changtai Zhu, Yawen Chen, Zhongyi Hu

**Affiliations:** 1 Shanghai Key Laboratory of Tuberculosis, Shanghai Pulmonary Hospital, Tongji University School of Medicine, Shanghai, China; 2 Department of Laboratory Medicine, Kunshan Hospital Affiliated to Nanjing University of Traditional Chinese Medicine, Kunshan, China; 3 Department of Nursing, Shanghai Jiao Tong University Affiliated Sixth People’s Hospital, Shanghai, China; 4 Department of Transfusion, Shanghai Jiao Tong University Affiliated Sixth People’s Hospital, Shanghai, China; Indian Institute of Technology Delhi, INDIA

## Abstract

*Mycobacterium tuberculosis* (MTB) is a specific aerobic bacterium, but can survive under hypoxic conditions, such as those in lung cheese necrosis, granulomas, or macrophages. It is not clear whether the drug sensitivity and growth characteristics of MTB under hypoxic conditions are different from those under aerobic conditions. In this study, we examined the drug resistance and growth characteristics of MTB clinical isolates by a large sample of *in vitro* drug susceptibility tests, using an automatic growth instrument. Under hypoxic conditions, variance in drug resistance was observed in nearly one-third of the MTB strains and was defined as MTB strains with changed drug sensitivity (MTB-CDS). Among these strains, resistance in a considerable proportion of clinical strains was significantly increased, and some strains emerged as multi-drug resistant. Growth test results revealed a high growth rate and large survival number in macrophages under hypoxia in MTB-CDS. According to the results of fluorescence quantitative PCR, the expression of some genes, including RegX3 (involving RIF resistance), Rv0194 (efflux pump gene), four genes related to transcription regulation (KstR, DosR, Rv0081 and WhiB3) and gene related to translation regulation (DATIN), were upregulated significantly under hypoxic conditions compared to that under aerobic conditions (*p* < 0.05). Thus, we concluded that some MTB clinical isolates can survive under hypoxic conditions and their resistance could change. As for poor clinical outcomes in patients, based on routine drug susceptibility testing, drug susceptibility tests for tuberculosis under hypoxic conditions should also be recommended. However, the detailed mechanisms of the effect of hypoxia on drug sensitivity and growth characteristics of MTB clinical isolates still requires further study.

## Introduction

Tuberculosis (TB) is a serious health problem, and China is one of the countries with high burden of TB [1,2]. One of causes for the difficulty in controlling tuberculosis is TB drug resistance [3–6]. In recent years, there has been a rising trend in the number of multi-drug resistant *Mycobacterium tuberculosis* (MTB) clinical isolates [5–9].

Currently, to guide the clinically rational use of drugs, routine *in vitro* drug susceptibility tests for TB are commonly conducted in many laboratories [10–13]. However, despite therapy based on the test results, there are still some cases of treatment failure [14]. We speculate that one of the causes for this may be related to changes in MTB resistance under hypoxia.

The routine TB drug susceptibility tests in vitro are performed under aerobic conditions (21% of oxygen from the atmosphere). However, in vivo conditions such as granuloma, caseous necrosis tissue, or macrophages are hypoxic in nature [15–17]. If MTB resides in these tissues, the features of its biological metabolism might change [15–18], which may lead to variation in its resistance and growth characteristics. To identify whether the drug sensitivity and growth characteristics of MTB under hypoxic conditions are different from those under aerobic conditions, and to explore the related mechanisms, we conducted the following related research, using a large sample of MTB clinical isolates.

## Methods and Materials

### Ethics statement

This study was approved by the Shanghai Pulmonary Hospital Affiliated to Tongji University School of Medicine Ethics Committee. Subjects were treated in accordance with the Helsinki Declaration on the participation of human subjects in medical research. Written informed consent was obtained from each participant.

### Collection of strains and drug susceptibility testing

Two hundred and forty-five MTB clinical isolates were obtained from the Department of Clinical Laboratory Medicine at Shanghai Pulmonary Hospital between 2013 and 2015 (S1 Fig). Routine drug susceptibility tests were performed using a commercial microplate kit (Yibaishi Biotech, Inc., Shen-zhen, China) on the BACTECT 960 System (Becton Dickinson, Franklin Lakes, NJ) under conventional aerobic conditions, and hypoxic drug susceptibility testing was conducted under hypoxic conditions by covering 50 μl of paraffin oil. In this study, susceptibility to first-line drugs like isoniazid (INH), ethambutol (EMB), rifampicin (RMP), and streptomycin (SM) was analyzed. The minimum inhibitory concentrations (MICs) were recorded. The standard laboratory strain H37Rv was purchased from the Chinese National Institute for Food and Drug Control.

### Establishment of the hypoxia model and analysis of growth characteristics of MTB

Aerobic cultures (NRP-2) by Wayne's method were used to establish the hypoxia model described in this report [15,19]. Population growth curves were determined by a Bioscreen Growth Curve Instrument (Bioscreen C, Helsinki, Finland), using a honeycomb plate with 100 wells (Bioscreen C, Helsinki, Finland). Briefly, 300 μl of 7H9 medium was added to each well, and cultured with shaking at 37°C. The optical density was measured as absorbance at 600 nm after every 2 hours. Hypoxia conditions were established by covering 50 μl of paraffin oil. If both growth curves under the aerobic and hypoxic conditions were completely in accord with the Webster's model, the model of hypoxia was considered successful. The growth characteristics analysis was performed under aerobic as well as hypoxic conditions.

### Enumerating MTB clinical strains in macrophages

Clinical isolates of MTB were cultured under both aerobic and hypoxic conditions. MTB isolates grown to the logarithmic phase were used to infect THP1 macrophages. At different time points after infection, the infected cells were washed twice with PBS and lysed for 10 minutes using Triton X-100. Subsequently, the lysed mixture was inoculated onto 7H9 solid plates and cultured at 37°C for 4 weeks. Finally, MTB clones were counted.

### Effect of hypoxia on gene expression in MTB clinical isolates

After reviewing previous studies [11,15–43], the relevant regulatory genes for efflux pumps and transcription factors likely to affect drug resistance in MTB (Table 1) were selected and their expression was quantified by RT-PCR. Briefly, 25 ml of culture was used to extract RNA upon cell lysis via the TRIzol bead beater method and phenol extraction [35]. RNA concentrations were determined using a Nanodrop 2000 (NanoDrop Technologies). RNA was treated with DNase as previously described [35] and reverse-transcribed using the High Capacity RNA-to-cDNA kit (Applied Biosystems) following the manufacturers’ instructions. RT PCR was performed using Power SYBR Green PCR Master Mix (Applied Biosystems), as previously described [35]. PCR primers for the above genes are specified in S1 Table. The tests in this study were performed on the 7500 Fast Dx Real-Time PCR System (Applied Biosystems). The reaction conditions were as follows: 95°C for 60 s, 40 cycles of 95°C for 5 s, 62°C for 8 s, and 72°C for 20 s, followed by a melt curve analysis.

**Table 1:** The characteristics of the testing genes in this study.

Category	Gene name	Rv number	Description
INH regulation gene	FbpC	Rv0129c	Diacylglycerol acyltransferase/mycolyltransferase Ag85C
INH regulation gene	EfpA	Rv2846c	MFS-type transporter EfpA
INH regulation gene	FadE23	Rv3140	Acyl-CoA dehydrogenase FadE23
RIF regulation gene	RegX3	Rv0491	Two component sensory transduction protein RegX
RIF regulation gene	SigF	Rv3286c	RNA polymerase sigma factor SigF
RIF regulation gene	MprA	Rv0981	Two-component response regulator MrpA
Efflux pump regulation gene	Rv0194	Rv0194	Multidrug ABC transporter ATPase/permease
Efflux pump regulation gene	WhiB7	Rv3197A	Transcriptional regulator WhiB7
Efflux pump regulation gene	Rv2136c	Rv2136c	Undecaprenyl-diphosphatase
Efflux pump regulation gene	Rv1410c	Rv1410c	Aminoglycosides/tetracycline-transport integral membrane protein
Transcript regulation gene	Rv0081	Rv0081	HTH-type transcriptional regulator
Transcript regulation gene	Rv0324	Rv0324	Transcriptional regulator
Transcript regulation gene	LipY	Rv3097c	Triacylglycerol lipase Lip
Transcript regulation gene	LipX	Rv1169c	Lipase LipX
Transcript regulation gene	Pks3	Rv1180	Polyketide beta-ketoacyl synthase
Transcript regulation gene	MutA	Rv1492	Methylmalonyl-CoA mutase small subunit
Transcript regulation gene	AccA3	Rv3285	Bifunctional protein acetyl-/propionyl-CoA carboxylase subunit alpha AccA
Transcript regulation gene	WhiB3	Rv3416	Redox-responsive transcriptional regulator WhiB3
Transcript regulation gene	KstR	Rv3574	HTH-type transcriptional regulator KstR
Transcript regulation gene	Lsr2	Rv3597c	Iron-regulated H-NS-like protein
Transcript regulation gene	DevR	Rv3133c	Two component transcriptional regulator dosR
Transcript regulation gene	IciA	Rv1985c	HTH-type transcriptional regulator
Transcript regulation gene	DATIN	Rv0079	Dormancy Associated Translation Inhibitor

### Statistical analysis

SPSS software (SPSS Inc., Chicago, IL, USA) was used for the statistical analysis of results. Chi-square test, non-parametric test, and one-way analysis of variance (ANOVA) were performed to analyze the different data in this study. A p-value less than 0.05 was considered statistically significant.

## Results

### Drug resistance of clinical strains under hypoxic conditions

In this study, the MICs of 245 MTB clinical isolates determined by the microplate method were found to be identical with those obtained by the BACTECT 960 System (S2 Table).

Addition of liquid paraffin to the microplate allowed the formation of a curve similar to that in the Wayne’s aerobic model, as determined by OD (S2 Fig). Therefore, construction of a hypoxic growth model in the microplate was successful.

**Fig 1:**
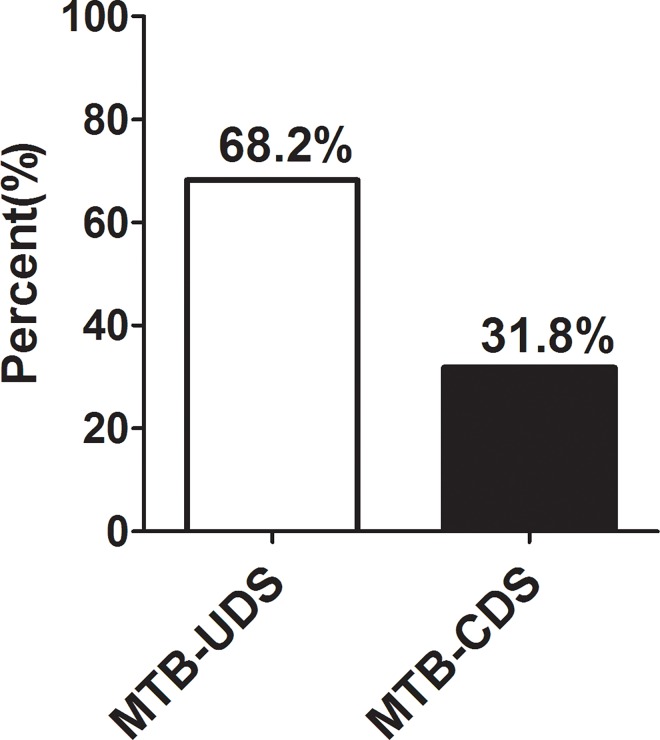
Comparison of the rates of changed MTB strains of drug sensitivity and unchanged MTB strains of drug sensitivity in 245 strains clinical isolates under hypoxia. MTB-UDS: changed MTB strains of drug sensitivity; MTB-CDS: unchanged MTB strains of drug sensitivity.

Upon analyzing the MICs of 245 clinical isolates, 68.2% (167/245) were determined as MTB strains of unchanged drug sensitivity (MTB-UDS), whereas others (31.8%, 78/245) belonged to MTB strains of changed drug sensitivity (MTB-CDS; Fig 1).

**Fig 2:**
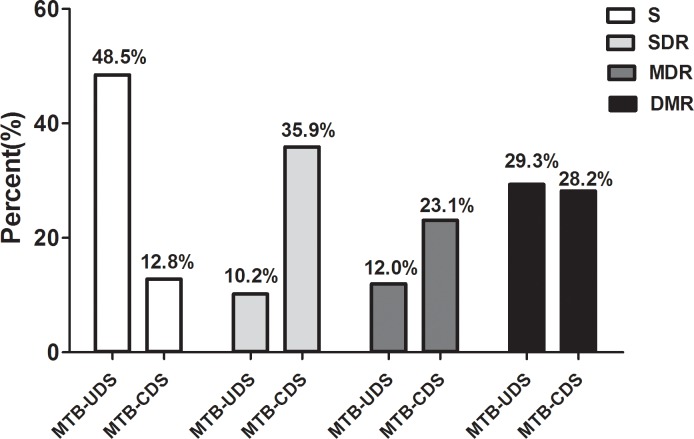
Comparison of drug resistances of changed MTB strains of drug sensitivity and unchanged MTB strains of drug sensitivity. MTB-UDS: changed MTB strains of drug sensitivity under hypoxia; MTB-CDS: unchanged MTB strains of drug sensitivity under hypoxia; S: sensitive; SDR: single drug resistance; MDR: multi-drug resistance; DMR: drug multi-resistance.

The stratified analysis of the drug susceptibility test result showed that the overall sensitivity in MTB-CDS was significantly lower than that in MTB-UDS (12.8% *vs*. 48.5%, *p* < 0.05). Resistance to single drugs in MTB-CDS was also significantly higher than that in MTB-CDS (35.9% *vs*. 10.2%, *p* < 0.05); multi-drug resistance in MTB-CDS was also significantly higher than that in MTB-UDS (23.1% *vs*. 12%, *p* < 0.05; Fig 2).

Overall, under hypoxic conditions, the rate of MTB clinical isolates for which the MICs of first line drugs were increased was 38.5% (30/78), whereas that for which the MICs were decreased was 23% (18/78; S3 Fig).

### Growth characteristics of MTB clinical isolates under hypoxic conditions

**Fig 3:**
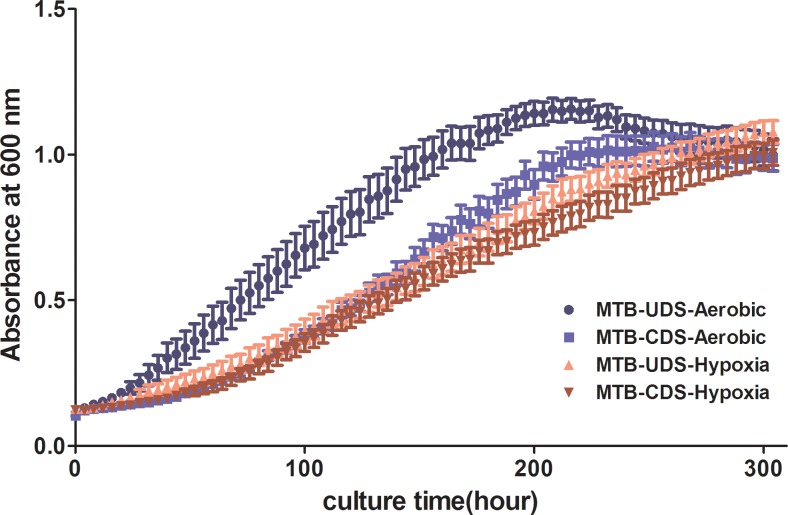
Growth curves of MTB clinical isolates under aerobic and hypoxic conditions. MTB-UDS: changed MTB strains of drug sensitivity; MTB-CDS: unchanged MTB strains of drug sensitivity.

Based on the growth curve analysis, the growth of MTB-CDS was slower that of MTB-UDS under aerobic conditions; whereas under hypoxic conditions, the growth curves of MTB-CDS nearly coincided with those of MTB-UDS, (Fig 3) indicating that MTB-CDS can suitably grow under hypoxia.

### Analysis of survival ability of MTB clinical strains in macrophages under hypoxic conditions

**Fig 4:**
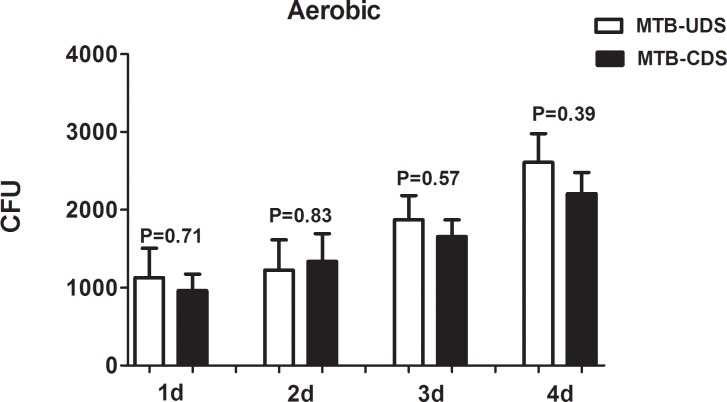
Survivability of changed MTB strains of drug sensitivity and unchanged MTB strains of drug sensitivity in macrophage cell under aerobic conditions. MTB-UDS: changed MTB strains of drug sensitivity; MTB-CDS: unchanged MTB strains of drug sensitivity.

**Fig 5:**
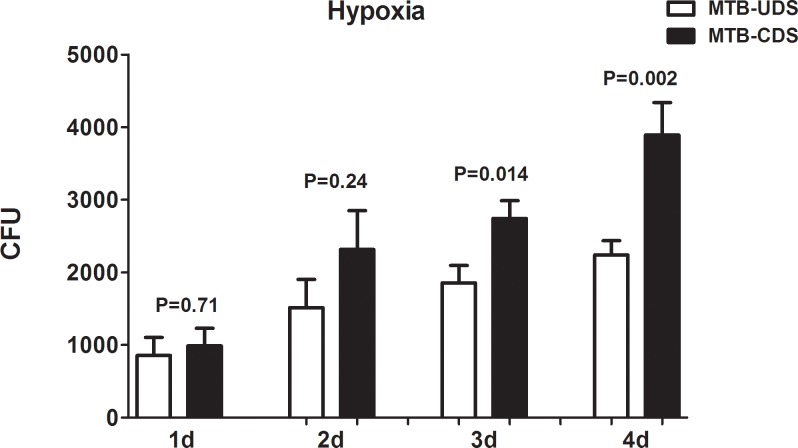
Survivability of changed MTB strains of drug sensitivity and unchanged MTB strains of drug sensitivity in macrophage cell under hypoxic conditions.

Examination of the MTB survival ability in macrophages at different times under aerobic conditions showed that the difference in survival between the MTB-CDS and MTB-UDS with increasing time was not statistically significant (*p* > 0.05; Fig 4); whereas under hypoxic conditions, the survival of the MTB-CDS was significantly higher than that of MTB-UDS (*p* < 0.05; Fig 5).

### Gene expression potentially related to resistance in MTB

According to the results of fluorescence quantitative PCR, the expression of some genes, including RegX3 (involving RIF resistance), Rv0194 (efflux pump gene), four genes related to transcription regulation (KstR, DosR, Rv0081 and WhiB3) and gene related to translation regulation (DATIN), was significantly upregulated under hypoxic conditions compared to that under aerobic conditions (*p* < 0.05; S4, S5, S6 and S7 Figs).

## Discussion

Previous studies have shown that MTB can survive under hypoxic conditions [23,44,45]. In this study, MICs of 245 MTB clinical isolates, determined by the microplate method, were found to be identical with those obtained from the BACTECT 960 System under aerobic conditions. The Wayne model is currently recognized as a reliable *in vitro* model for simulating *in vivo* environments such as MTB dormancy or hypoxic environments. In this study, we observed almost coinciding curves upon comparing the growth curve of the MTB culture systems with H37Rv, which was cultured under both aerobic and anaerobic environments. Therefore, the growth model of hypoxia was successfully established. By analyzing the MICs of 245 clinical isolates, we found that about one-third of the MTB strains were MTB-CDS, whereas the others belonged to MTB-UDS.

Further stratified analysis of the results of drug sensitivity testing showed that the overall sensitivity of MTB-CDS was significantly lower than that of MTB-UDS, and the resistance to single drugs and multiple drugs in MTB-CDS were both significantly higher than those in MTB-UDS. Overall, under hypoxic conditions, the proportion of MTB clinical isolates for which the MICs of first line drugs were increased was 38.5%.

Therefore, we can conclude that there was a significant change in drug resistance of some MTB strains under hypoxic conditions. Considering that routine drug susceptibility testing is unable to completely reflect the actual resistance in MTB, we suggest that drug susceptibility tests should also be conducted under hypoxic conditions to exclude hypoxia resistance in MTB, especially for cases showing initial therapeutic failure.

Based on the growth curve analysis, the growth of MTB-CDS was slower than that of MTB-UDS under aerobic conditions, whereas under hypoxic conditions, the growth curves of MTB-CDS were nearly similar to those of MTB-UDS, indicating that MTB-CDS are more suitable for survival under hypoxia.

By determining the survival of MTB in macrophages at different times, we found that the difference in survival under aerobic conditions was not statistically significant between the MTB-UDS and MTB-CDS with increasing time; however, under hypoxic conditions, the survival of MTB-UDS was significantly higher than that of MTB-CDS. This further proves that MTB-CDS can survive under hypoxic conditions.

A previous study has shown that the dormancy survival regulon (DosR regulon) is chiefly responsible for encoding the dormancy related functions of MTB, and appears to be involved in translation regulation through the interaction of its product with bacterial ribosomal subunits [46]. One of the DosR regulon proteins, DATIN, encoded by the gene Rv0079, can stimulate macrophages and peripheral blood mononuclear cells to secrete important cytokines, which may be significant in granuloma formation and maintenance [47]. Additionally, the expression of DATIN in *M*. *bovis* BCG was found to be upregulated under pH stress and micro-aerobic conditions [47]. This indicates that MTB survival under hypoxia is closely associated with gene regulation, and some relevant regulatory genes related to drug resistance, such as efflux pumps and transcription factors, are likely involved in MTB drug resistance under hypoxia. According to the results of fluorescence quantitative PCR, the expression of some genes, including RegX3 (involving RIF resistance), Rv0194 (efflux pump gene), four genes related to transcription regulation (KstR, DosR, Rv0081 and WhiB3) and gene related to translation regulation (DATIN), was significantly upregulated under hypoxic conditions compared to that under aerobic conditions. As for other genes such as IciA, we didn’t found difference in gene expression. However, the previous study showed that, IciA has a bearing on the functional role of the important regulator of MTB chromosomal replication in the context of latency [48]. According to the report, synergy between the N-terminal and C-terminal domains of HupB is essential for its DNA-binding ability, and to modulate the topological features of DNA, which has implications for processes such as DNA compaction, gene regulation, homologous recombination, and DNA repair [49]. Thus, we believe that the mechanisms of hypoxia resistance in MTB clinical isolates are associated with the expression of some genes. Moreover, we speculate that the difference in the expression of some genes could result in better survival of MTB-CDS under hypoxia compared to that of MTB-CDS.

Efflux pumps are thought to have emerged for the expulsion of noxious substances from the bacterial cell, thus allowing its survival; increased expression of efflux pumps is associated with resistance to alien substances, including drugs [50]. These pumps are often called multi-drug efflux pumps [50]. In addition, the expression of drug resistance in MTB is linked with some transcription regulation genes. However, the exact mechanisms involved in hypoxia drug resistance of MTB remain unknown.

In summary, in our study, some MTB clinical isolates showed very good adaptability under hypoxic conditions, better survival in macrophages, and resistance to some anti-tuberculosis drugs. This resulted in incorrect results from routine MTB drug susceptibility testing; hence, we recommend that drug susceptibility tests should be simultaneously conducted under hypoxic conditions, especially for cases with initial therapeutic failure. The mechanisms underlying the effect of hypoxia on drug sensitivity and growth characteristics of MTB still require further study.

## Supporting Information

S1 Fig Flow chart of the *M*. *tuberculosis* clinical isolates included in this study. (TIF)

S2 Fig Growth curves of MTB clinical isolates under aerobic or hypoxic conditions. (TIF)

S3 Fig Drug resistance characteristics of MTB clinical isolates under aerobic or hypoxic conditions. (TIF)

S4 Fig Expression of Rv0194 and RegX3 genes under aerobic or hypoxic conditions. (TIF)

S5 Fig Expression of KstR and WhiB3 genes under aerobic or hypoxic conditions. (TIF)

S6 Fig Expression of DosR and Rv0081 genes under aerobic or hypoxic conditions. (TIF)

S7 Fig Expression of DATIN genes under aerobic or hypoxic conditions. (TIF)

S1 Table PCR primers and conditions used in this study. (XLS)

S2 Table Overall drug resistance characteristics of the MTB clinical isolates. (XLS)
