# Supplementary figures and images for: Impact of Hypoxia on Drug Resistance and Growth Characteristics of Mycobacterium tuberculosis Clinical Isolates
Source: PLoS One. 2016 Nov 11;11(11):e0166052. doi: 10.1371/journal.pone.0166052 (PMC5106006; doi:10.1371/journal.pone.0166052)

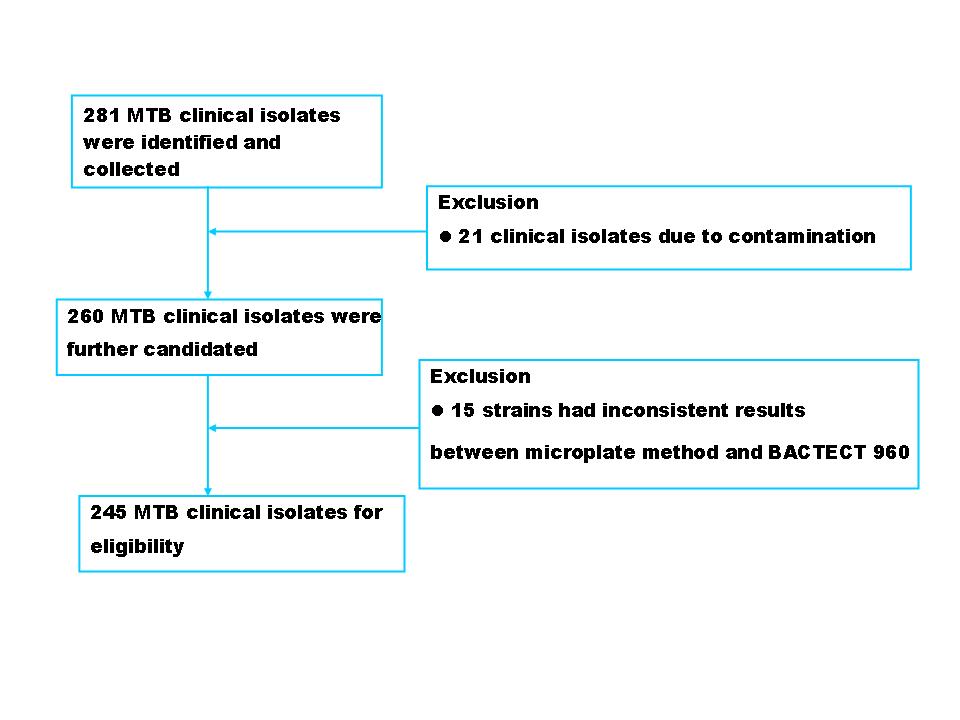

Supplement: S1 Fig — (TIF) [file pone.0166052.s001.tif]

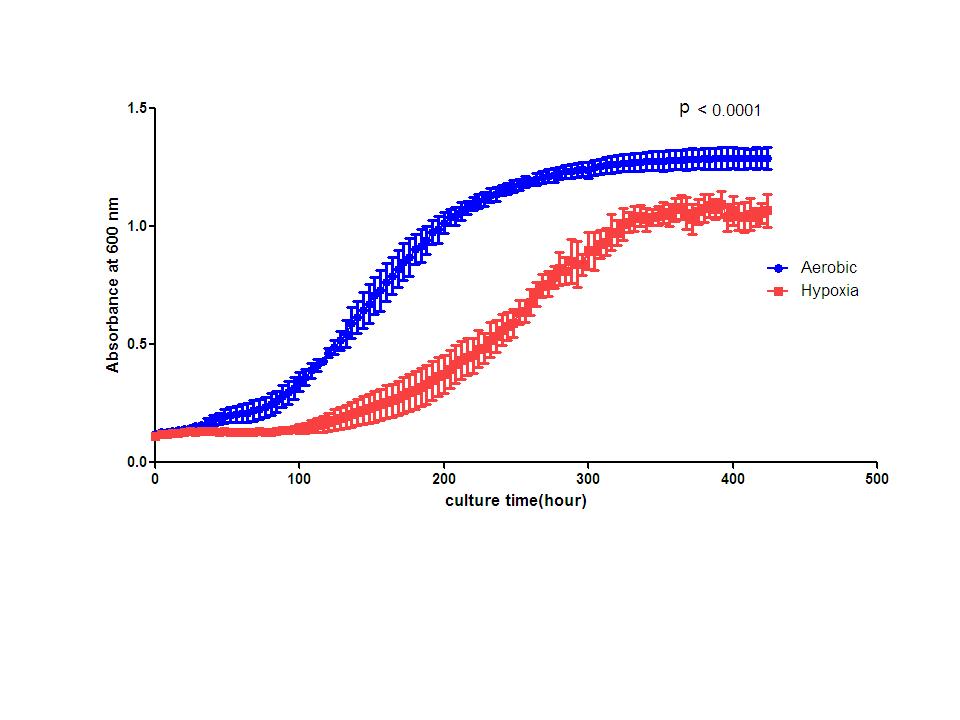

Supplement: S2 Fig — (TIF) [file pone.0166052.s002.tif]

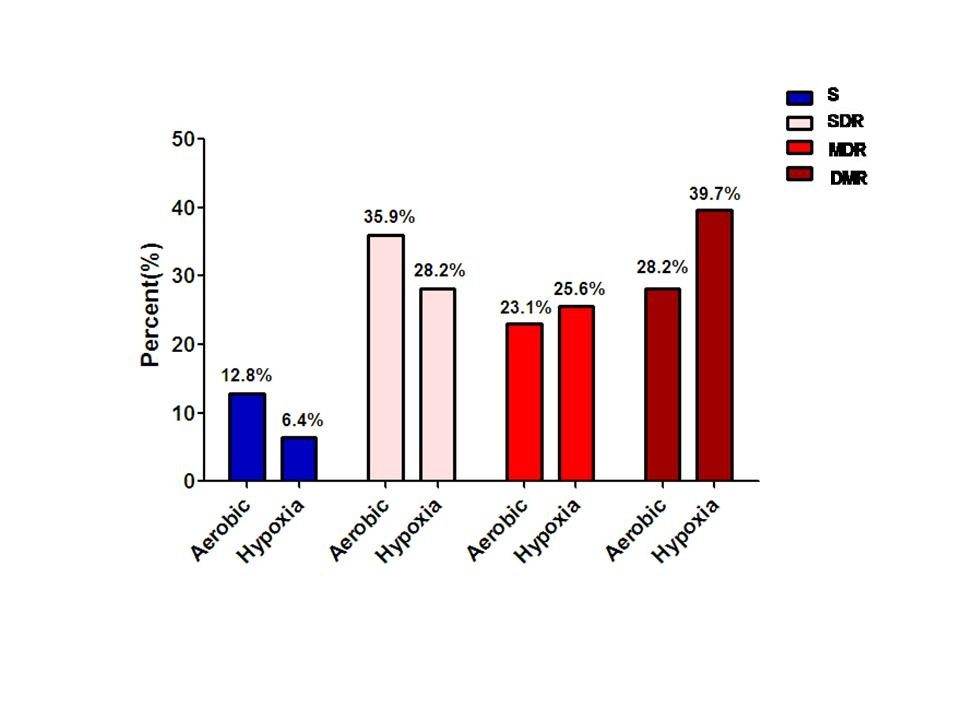

Supplement: S3 Fig — (TIF) [file pone.0166052.s003.tif]

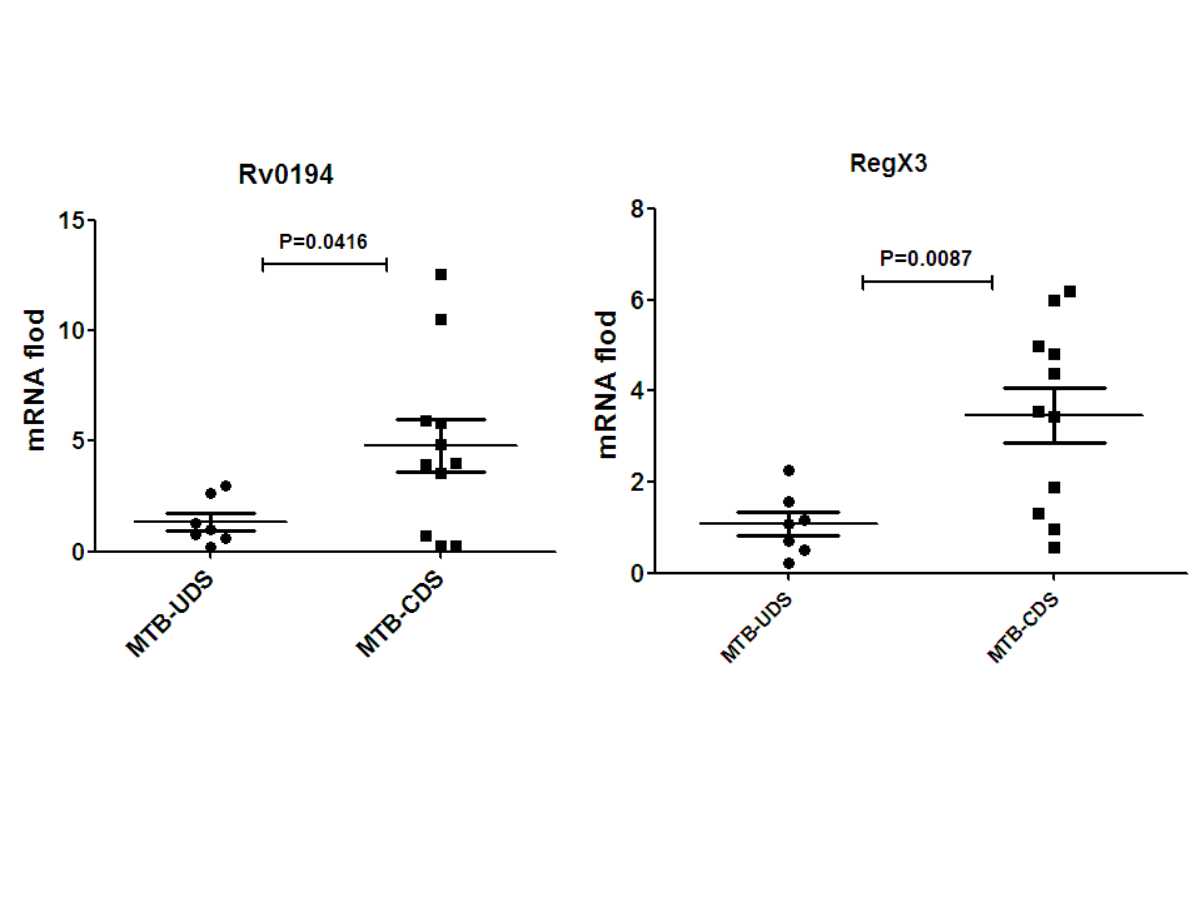

Supplement: S4 Fig — (TIF) [file pone.0166052.s004.tif]

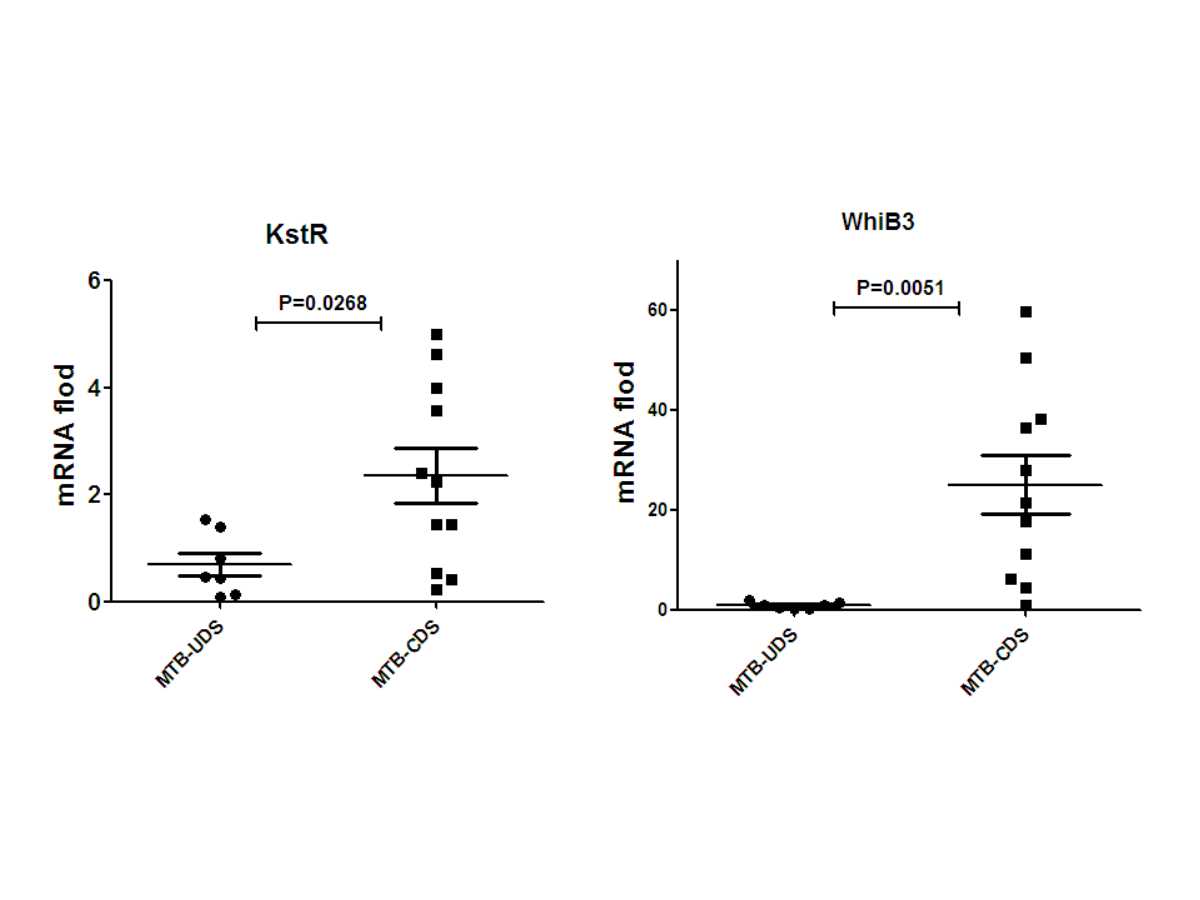

Supplement: S5 Fig — (TIF) [file pone.0166052.s005.tif]

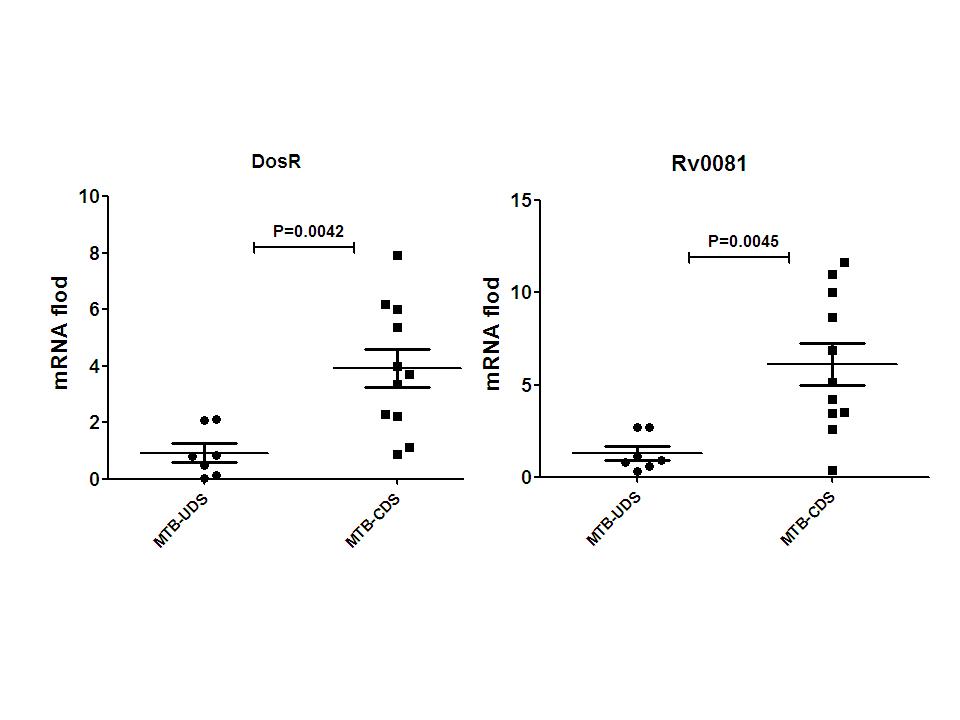

Supplement: S6 Fig — (TIF) [file pone.0166052.s006.tif]

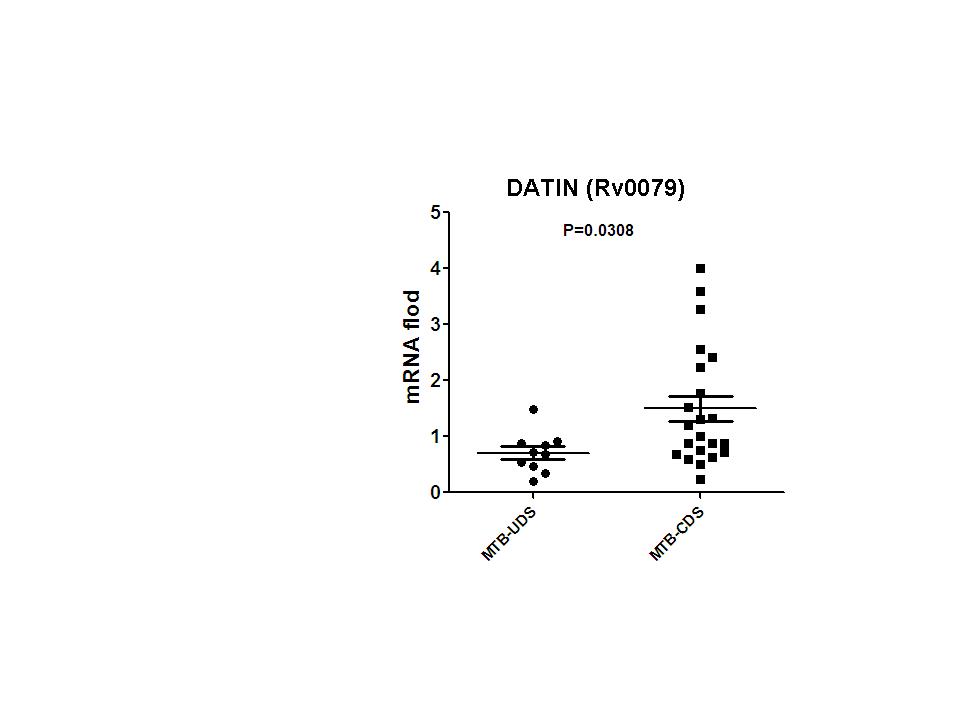

Supplement: S7 Fig — (TIF) [file pone.0166052.s007.tif]
